# A one-compartment model provides benchmark Lithium dose prediction

**DOI:** 10.1177/02698811251378508

**Published:** 2025-10-29

**Authors:** Oisín N. Kavanagh, Elliot Asprey, Katinka A. Edelmann, Philipp Ritter, David A. Cousins, Victoria C. Wing

**Affiliations:** 1 School of Pharmacy, Newcastle University, Newcastle upon Tyne, UK; 2 Translational and Clinical Research Institute, Faculty of Medical Sciences, Newcastle University, Newcastle upon Tyne, UK; 3 Department of Psychiatry and Psychotherapy, University Hospital Carl Gustav Carus, Technische Universität Dresden, Dresden, Germany; 4 Institute of Psychiatry, Psychology and Neuroscience, King’s College London, London, UK; 5 Newcastle Magnetic Resonance Centre, Health Innovation Neighbourhood, Newcastle University, Newcastle upon Tyne, UK; 6 Cumbria, Northumberland, Tyne and Wear (CNTW) NHS Foundation Trust, UK

**Keywords:** lithium, therapeutic drug monitoring, dose prediction, one-compartment model, pharmacokinetics

## Abstract

**Background:**

Lithium is an effective treatment for recurrent affective disorders, but it has a narrow therapeutic window and requires regular serum concentration monitoring, especially during periods of dose titration. Numerous attempts have been made to develop dose prediction methods to facilitate initiation and swift achievement of effective levels, but these typically lack sufficient accuracy and can be challenging to implement in practice.

**Aims:**

Develop a pharmacokinetic model of lithium to enable accurate dose prediction which is adaptable for clinical practice.

**Methods:**

The calculator was developed from a one-compartment model, which assumes that lithium distributes into total body water and requires only simple body measurements (age, sex, height and weight) as input variables. Its performance was compared to six commonly cited dose prediction methods in patients with bipolar disorder taking lithium, using two independent research samples from the United Kingdom (*n* = 40) and Germany (*n* = 18).

**Results:**

Our one-compartment model performed better than the previous models, accurately predicting the required lithium dose within one 200 mg lithium carbonate tablet. The mean prediction error was 10 mg (SD = 148 mg) in this sample of euthymic subjects taking stable doses of lithium sampled at steady state.

**Conclusions:**

This model sets a new benchmark for lithium dose prediction accuracy and requires only simple body measurements. Further validation work in larger, diverse samples and future developments, such as the ability of the model to back-calculate levels from samples taken outside the recommended 12-hour window, may support its translation and use in practice.

## Introduction

Mood disorders are debilitating psychiatric conditions associated with increased morbidity and premature mortality (McIntyre et al., 2020), as well as elevated risk of suicide (Dong et al., 2020). Since its discovery as an anti-manic agent over 70 years ago (Cade, 1949), lithium has accrued the best evidence base for prophylaxis in bipolar disorder, preventing episodes of illness (Cleare et al., 2015) and reducing suicidal behaviour (Smith and Cipriani, 2017). In major depression, it is an effective antidepressant augmentation agent, and lessens the risk of recurrence and hospitalisation with long-term use (Cleare et al., 2015). Despite these benefits, the use of lithium is in decline (Zivanovic, 2017), likely in part due to the demands of regular blood monitoring and concerns about toxicity and longer-term adverse effects.

Lithium has a narrow therapeutic window, between 0.4 and 1.0 mmol/L for most indications. Whilst toxicity can occur in the therapeutic range, it most commonly manifests with levels above 1.5 mmol/L and can be associated with severe health consequences (Murphy et al., 2023). Vigilance is also required to reduce the risk of adverse effects in the long-term, as even a single measurement above 1.0 mmol/L has been linked to a sustained reduction in kidney function (Kirkham et al., 2014). Conversely, failure to achieve adequate serum concentrations is associated with a greater risk of relapse (Hsu et al., 2021), such that a range of 0.6 to 0.8 mmol/L is commonly advocated in practice and by expert consensus in bipolar disorder (Nolen et al., 2019). In practice, the dose required to achieve therapeutic concentrations for a given patient is usually reached by titration, typically guided by weekly blood tests until stable levels in the desired range are achieved (British National Formulary, 2025). Clinical judgement is typically exercised, with dose increments guided by factors such as patient age, co-morbid conditions, concurrent medications and the severity of the presentation requiring lithium. This is arguably best achieved by clinicians who are familiar with lithium initiation, avoiding extended periods until optimal treatment is established, and engaging patients to demonstrate that monitoring can be readily undertaken. Tools to support this process, such as the ability to predict the required dose for a given patient and plan titration, may reverse the decline in lithium use, ultimately to the benefit of those with significant mood disorders.

Lithium dosage prediction has a long and varied history, with at least 38 methods reported in over 65 publications in the last 50 years. The approaches can be broadly split into ‘first dose’ methods (which require a serum lithium measurement after one dose) and *a priori* methods (predicting dose from baseline characteristics; Sienaert et al., 2013). Whilst there are still fundamental questions about the pharmacokinetics of lithium that remain unanswered, many dose prediction models do not factor in such parameters and instead employ statistical techniques such as linear regression analyses, often derived in small populations. Methods that do integrate pharmacokinetic parameters typically select surrogate measures, such as the estimated glomerular filtration rate (eGFR), to approximate lithium elimination. Further, due to the nature of their construction, many models do not allow clinically important parameters to be factored in, such as the sampling time, dosage regimen (once vs. twice daily), formulation differences, and the desired serum lithium concentration. More recently, population pharmacokinetic (popPK) approaches have been employed to better describe lithium pharmacokinetics, but such models can struggle to extrapolate their findings across diverse patient populations due to high interindividual variability and the influence of difficult-to-quantify factors like renal elimination.

In response to these challenges, we present the development of a one-compartment model that can be adjusted for dosing parameters and uses simple demographic and body measurements (age, sex, height and weight) to predict lithium serum concentrations, to calculate a lithium dose *a priori*. From the pre-existing models identified in a systematic review (Sienaert et al., 2013) we compared our calculator against six commonly cited *a priori* methods ((Keck Jr et al., 2001), (Zetin et al., 1986), (Chiu et al., 1976), (Stip et al., 2001), (Terao et al., 1995) and (Abou-Auda et al., 2008)), as well as a population PK model, using the data from two research cohorts of euthymic patients with bipolar disorder taking a stable dose of lithium.

## Methods

### Patient data collection

Data was made available from patients with bipolar disorder who had enrolled in two separate observational lithium magnetic resonance imaging (^7^Li-MRI) studies (imaging data was not used in the development or testing of the model). The Bipolar Lithium Imaging and Spectroscopy Study (BLISS) was conducted at Newcastle University, UK (Smith et al., 2018) and the Temporospatial lithium CNS kinetics in Bipolar Disorder using repeat scan ^7^Li-MRI brain imaging study (BIPOLITH) at University Hospital Dresden, Germany. In both studies, subjects were included if they had a diagnosis of bipolar disorder (type I or II), were euthymic in mood, and were taking lithium as a long-term treatment at a stable dose with a once-daily regimen.

In the BLISS cohort (*n* = 40), subjects were recruited from primary and secondary care services in the North of England, and the diagnosis of bipolar disorder (type I or II) was confirmed through clinical interview using the NetSCID diagnostic tool (Structured Clinical Interview for DSM-V Disorders; SCID-5-CV version). Euthymic mood state was defined as scores of <7 on both the 21-item Hamilton Depression Rating Scale and the Young Mania Rating Scale. Exclusion criteria were contraindications to MRI examination, current or recent harmful drug or alcohol use, comorbid diagnosis, learning disability, impairment of capacity or current liability to detention under the Mental Health Act 1983 (amended 2007). All subjects had taken lithium carbonate once daily regularly for at least one year, and concomitant use of other psychotropic medication was permitted at inclusion. Prior to the study visit, where lithium levels were taken at 09.00, subjects were instructed to take their lithium as usual the night before.

The BIPOLITH study sought to compare brain lithium concentrations in once versus twice daily dose regimens, but data were only used from subjects taking lithium once daily (*n* = 19). Euthymic subjects with bipolar disorder (type I or II) were recruited from primary and secondary health care services in Germany and underwent a screening visit to confirm diagnosis through clinical interview using the SCID-5-CV. Subjects were excluded if they had contraindications to MRI examination and/or severe medical conditions incompatible with subject adherence to study protocol, including but not limited to diabetes, epilepsy, uncontrolled hypertension, and renal impairment (GFR ⩽ 60). Participants were instructed to take their lithium dose 12 hours prior to their appointment, where a sample was drawn.

### Ethics statement

The authors assert that all procedures contributing to this work comply with the ethical standards of the relevant national and institutional committees on human experimentation and with the Helsinki Declaration of 1975, as revised in 2013. All subjects provided written informed consent, including further use of their data for bona fide research, and the studies were approved by a National Research Ethics Committee (BLISS, 14/NE/1135; BIPOLITH, EK86022019).

### Constructing a one-compartment model

Key pharmacokinetic parameters were extracted from the product literature and the small number of studies available to construct our one-compartment model. The bioavailability of lithium carbonate (*F* = 90%) was determined by taking the ratio of the Area Under the Curve of 13.5 mmol lithium absorbed from oral (6.44 mM.h) (Guelen et al., 1992) and IV (7.13 mM.h) (Waring et al., 2002) formulations. The half-life of lithium was taken to be 24 hours (*k* = 0.0289), an average value from 16 to 32 hours according to product information. (Electronic Medicines Compendium, 2025)

The Pepin formula (Stip et al., 2001), is derived from classical one-compartment modelling equations (Equation 1; Rowland and Tozer, 1994), but we departed from this as our early explorations revealed a systematic underestimation of the lithium dose. Developing the assumption that lithium at steady state distributes into total body water (TBW; Amdisen, 1978; ElDesoky et al., 2008), we employed an estimate of TBW for individual patients (Watson et al., 1980), and set this to equate to the volume of distribution at steady state. This approach formed the basis of our low error predictive model (Equation 2).

(1) $$C_{t}=\mathrm{Dose}\times \;e^{-k\;\times \;t}$$

(2) $$C_{t}=\frac{[Li]\times F}{\mathrm{Total}\;\mathrm{body}\;\mathrm{water}}\;\times \;e^{-0.0289\;\;\times \;t}$$

C is the concentration of lithium; t is the time post dose when the sample is withdrawn; [Li] is the amount in millimoles of lithium administered; F is the bioavailability of lithium (90%); the TBW is calculated from the Watson, Watson and Batt equation, provided below (Watson et al., 1980). These TBW equations were developed using linear regression compared to the gold-standard deuterium oxide dilution method in healthy volunteers. Separate equations best fit the data in males and females with weight and height included as variables in both, and age only in males.

$$\begin{array}{l} TBW_{\mathrm{male}}=2.447-(0.09516\times \mathrm{age})\\ \;\;\;\;\;\;\;\;\;\;\;\;\;\;\;\;\;\;\;\;\;+(0.1074\times \mathrm{height})+(0.3362\times \mathrm{weight}) \end{array}$$

$$TBW_{\mathrm{female}}=2.097-(0.1069\times \mathrm{height})+(0.2466\times \mathrm{weight})$$

To calculate the concentration of lithium at steady state, we employed Equations 3 and 4, where τ is the dosing interval.

(3) $$C_{\max,\;\mathrm{steady}\;\mathrm{state}}\;=\;\frac{\frac{[Li]\;\times \;F}{\mathrm{Total}\;\mathrm{body}\;\mathrm{water}}}{1-e^{-0.0289\;\times \;\tau }}$$

(4) $$C_{\min,\;\mathrm{steady}\;\mathrm{state}}=C_{\max,\;\mathrm{steady}\;\mathrm{state}}\times e^{-0.0289\;\times \;t}$$

Replacing *C_t_* (1) with *C*_min, ss_ (4), we were able to rearrange to find the lithium dose required for a given patient at steady state (in millimoles), producing Equation 5, which was evaluated in this study.

(5) $$[Li]=\frac{C_{\min,\mathrm{ss}}\times \mathrm{Total}\;\mathrm{body}\;\mathrm{water}}{F\;\times \;e^{-0.0289\;\times \;t}}$$

### *Constructing a* popPK *model of lithium*

We constructed a basic mixed-effect pharmacokinetic model using Monolix (v.2024, Lixoft. Antony, France), assuming that administration is extravascular with a first-order absorption (rate constant k_a_). The PK model had a central compartment (volume *V*) and a linear elimination (elimination rate *k*). We included a proportional error model and random effects on all the model parameters with *k_a_* = 1, *V_d_* = 45, and *k* = 0.028 used as initial estimates.

This model had large shrinkage (shrinking seeks to reduce variability and prevent overfitting). However, we deliberately employed this large shrinkage model because we wanted to compare our new one-compartment model (which should better account for intra-individual differences) to the best fit for the population, obtained via mixed-effects models, to provide an overall picture of lithium serum concentrations in the population. We employed the BLISS dataset to create our model as it included covariates that are often considered important in lithium pharmacokinetics. Specifically, serum creatinine measurements were available, which were used to calculate creatinine clearance as a means to compare our model against commonly cited *a priori* methods.

### Predictive value analysis

To assess the clinical utility of the model in identifying therapeutic lithium levels, we calculated the positive predictive value (PPV) and negative predictive value (NPV) based on binary classification of predicted and observed serum lithium concentrations. Predictions were categorised as ‘positive’ when the model output fell within a defined therapeutic range (0.6–1.0 mmol/L, which was determined as the suitable range for this group of working-age adults) and ‘negative’ when outside this range. These were compared to the corresponding observed serum lithium levels, which served as the reference standard. PPV was defined as the proportion of positive predictions that corresponded to observed therapeutic levels, while NPV was defined as the proportion of negative predictions that corresponded to observed non-therapeutic levels.

## Results

### Patient characteristics

Iterative analysis of the *z*-score of predictive error for our one-compartment model within each cohort identified outliers, which were excluded from the analysis (*z*-score threshold > 2.5), four from BLISS and one from BIPOLITH. This provided 36 and 18 subjects in the BLISS and BIPOLITH cohorts, respectively (combined *n* = 54). The demographic and clinical characteristics, as well as lithium doses taken in once-daily regimens, are shown in Table 1.

**Table 1.** Demographic and clinical characteristics of the BLISS (*n* = 36) and BIPOLITH (*n* = 18) cohorts after exclusion.

	BLISS	BIPOLITH
Characteristics	Value (Mean ± SD)	Value (Mean ± SD)
Sex (female:male)	20/16	10/8
Age (years)	51 ± 11	40 ± 10
Height (cm)	170 ± 10	175 ± 11
Weight (kg)	85 ± 16	81 ± 20
BMI	29 ± 5	26 ± 6
Lithium dose (mg/day)	794 ± 220	846 ± 230
Creatinine clearance (mL/min)*	113 ± 35	–
Serum lithium (mmol/L)	0.75 ± 0.14	0.69 ± 0.13

*Note.* BLISS = Bipolar Lithium Imaging and Spectroscopy Study.

* Estimated using the Cockcroft-Gault equation.

### Comparison of existing dose prediction methods with the one-compartment model

Using the BLISS dataset, we examined the performance of our proposed model against six commonly used *a priori* lithium prediction formulas (Table 2). We found that all previous methods had a mean error of greater than one 200 mg tablet when rounded to the nearest 200 mg, the smallest whole tablet of lithium carbonate available in the United Kingdom, and most had large standard deviations of up to three 200 mg tablets from the mean. Of the *a priori* approaches, Pepin’s one-compartment model had the lowest mean error and narrowest standard deviation, although its use in this population would have still underdosed patients by an average of one to two tablets. Our model produced a mean error of 53.7 mg (*SD* = 142 mg) in the BLISS cohort, and −59.4 mg (*SD* = 148 mg) in BIPOLITH. Combining both cohorts provided a mean error of 10.3 mg (SD = 148 mg).

**Table 2.** Performance of existing lithium prediction models were tested using BLISS exclusively (*n* = 36). BLISS and BIPOLITH data were *y* combined to test the model arising from this work (combined total, *n* = 54).

Method	Factors included	Mean prediction error ± Standard Deviation	
		Lithium carbonate (mg)	Rounded to the nearest whole 200 mg tablet of lithium carbonate
Keck	Weight	901 ± 299	5 ± 1
Chiu	Body weight Creatinine clearance Desired lithium concentration	693 ± 626	3 ± 3
Abou-Auda	Ideal body weight Creatinine clearance Desired lithium concentration	307 ± 210	2 ± 1
Zetin	Weight Age Hospitalization Gender Tricyclic antidepressant use Desired lithium concentration	−327 ± 211	−2 ± 1
Pepin	mmol Li in formulation Weight Height Time of plasma sample	−284 ± 102	−1 ± 1
Terao	Desired lithium concentration Blood urea nitrogen Age Weight	145 ± 173	1 ± 1
**This work**	**mmol Li in formulation** Weight Height Sex Age Time of serum sample	**10** ± **148**	**0** ± **1**

### Predictive value analysis

Our model demonstrates a high PPV of 0.87, indicating that when it predicts a therapeutic serum lithium level, it is correct approximately 87% of the time. In contrast, the NPV is considerably lower, at 0.125, meaning that when the model predicts a subtherapeutic or out-of-range level, it is only correct around 12.5% of the time.

### Understanding the predictive variability

**Figure 1.**
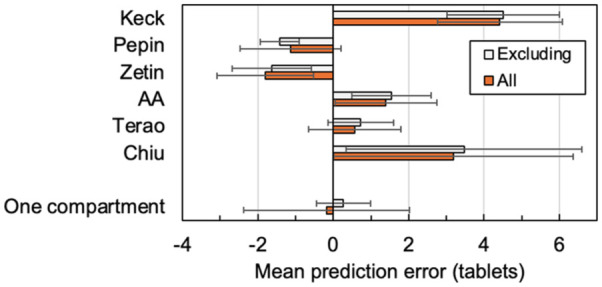
Model comparison including (orange, *n* = 40) and excluding (grey, *n* = 36) BLISS participants A, B, C and D.

We examined the characteristics of the four BLISS subjects (A, B, C and D) and one BIPOLITH (subject E) removed from our analysis because they produced the highest mean error for all models (e.g. error ⩾ 40% off target for the one-compartment model) and identified as outliers in our dataset (*z*-scores ⩾ 2.5). Subject C was prescribed 1400 mg of lithium and their 12-hour level was 0.3 mmol/L, potentially indicating that this patient did not take their dose within 12 hours of sampling. Conversely, subject B had a lithium dose of 400 mg but a level of 1.0 mmol/L, suggesting they may have taken their dose closer to the sampling window. Subjects A and D had the highest and lowest serum lithium levels in the dataset (1.0 and 0.24 mmol/L, respectively), with A receiving the largest lithium dose in the sample (1800 mg) and D one of the lowest with 400 mg. Subject E received a relatively large dose (1125 mg) and returned a serum lithium of 0.52 mmol/L (prediction 1.16 mmol/L). In brief, it is plausible that this group of patients deviated from the prescribed treatment plan by dose or timing. Removal of these participants had little effect on the performance of most of the other models, but did impact the one-compartment models (Pepin and this work, Figure 1). An F-test confirms that only Pepin, Terao and our model had a statistically significant reduction (*p* < 0.05) in variability after participant removal.

**Figure 2.**
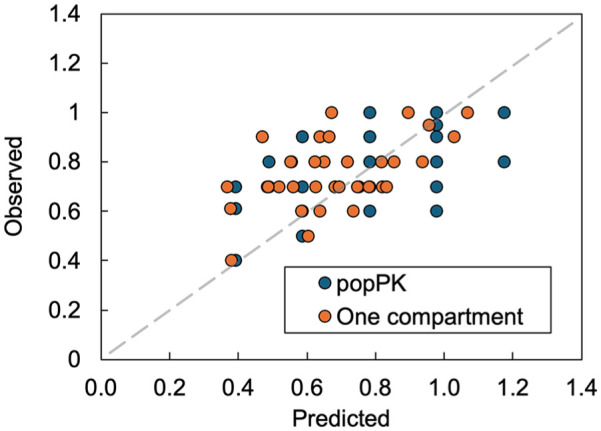
Comparison of predictions for one-compartment and popPK models using BLISS data. *Note.* BLISS = Bipolar Lithium Imaging and Spectroscopy Study; popPK = population pharmacokinetic.

While our proposed one-compartment model requires facile inputs and the mean predictive error for both cohorts is low, the standard deviation from the mean was almost one tablet (after exclusion of four participants from BLISS), which we felt could be optimised. We initially thought to explain this error due to missing covariates or unaccounted inter-individual differences but found no clear relationship between predictive error and weight, height, age, dose, serum creatinine, creatinine clearance and sex (Figure S1). Further exploration was facilitated by developing a popPK model using a linear mixed-effects approach. This model allowed for both fixed effects (to test for potential missing covariates) and random effects (to account for systematic inter-patient variability). This model was built using the BLISS dataset exclusively as it contained more covariates. In our final popPK model, we achieved a prediction error of −0.02 (SD = 0.19), which corresponded to −72.4 mg (SD  209 mg), similar to the performance of our one-compartment model in this cohort (53.7 mg SD = 142 mg, Figure 2).

## Discussion

We have derived a simple pharmacokinetic equation that can predict serum lithium levels in patients with much greater accuracy than previously reported *a priori* approaches. Our approach provides a means to identify a target lithium dose for a given patient with easily obtained clinical information (age, sex, weight and height) for samples taken at steady state and at a constant dose.

We employed first principles pharmacokinetic approaches over tools that derive lithium doses from linear regression because they can better accommodate inter-individual differences. They enable dose optimisation based on patient-specific covariates and facilitate the design of efficient clinical trials by reducing the need for extensive sampling. Recently, popPK models have attempted to capture variability in drug response across real-world patients to support therapeutic drug monitoring and personalised medicine approaches, and have been used in this context for lithium (Methaneethorn, 2018). However, popPK models can be limited by the representativeness of the study population. If the sampled population does not adequately reflect the target clinical population, model predictions may lack generalisability, leading to inaccurate dosing recommendations. Additionally, heterogeneity within the population, such as unrecognised subgroups or varying covariate effects, can complicate model development and reduce predictive accuracy. While linear regression has been used many times in the past to produce tools that may guide clinical decisions (Cockcroft and Gault, 1976; DuBois and DuBois, 1915), these models are vulnerable to losing their predictive capacity when applied to new populations. This has resulted in a variety of cautionary tales in the literature, proposing new equations to account for population differences, as predictions will always be skewed towards the original population. In other words, formulae produced from linear regression will only maintain accuracy if the population or patient being tested reflects the original population used to develop the model. This limitation was apparent when testing existing dose prediction methods in our sample, including the four developed from linear regression, which showed poor validity.

Pepin (Stip et al., 2001) was the first to apply a one-compartment model to lithium dose prediction, defining the volume of distribution using the population measurement, which led to a systematic underprediction of the dose by close to 300 mg in our analysis. Others have attempted to modify the Pepin formula to compensate for this error without success (Stip et al., 2001). As the lithium volume of distribution is quoted in the clinical literature as 0.7 L/kg (Electronic Medicines Compendium, 2025), we instead supposed that its volume of distribution may be explained by TBW, leading to a substantial reduction in error. To find an adaptable patient-specific estimate of TBW, we employed the much-cited Watson formula (Watson et al., 1980). Although this equation was derived by linear regression from a healthy American population (Chumlea et al., 2001), studies that have set out to examine the effect of racial origin on its accuracy have shown it performs comparably in African/Afro-caribbean (Davenport et al., 2011) and Korean populations (Lee et al., 2001). However, deviations can be expected in patients with altered fluid status or co-morbid conditions such as renal failure (Cooper et al., 2000; Noori et al., 2018). For example, in patients on peritoneal dialysis, one study suggested that caution should be exercised in the use of the Watson formula in very lean and obese patients, where predictions can be expected to deviate by up to 10 L as the ratio between TBW and body weight deviates ±0.2 from 0.5 (Johansson et al., 2001). Other rapid TBW estimation techniques, such as bioimpedance have been used, highlighting problems with the Watson formula for TBW predictions in some groups (Powers et al., 2009), but this technique also relies on linear regression models, which have the same pitfalls as standard anthropometric tools (Matias et al., 2016). Further research is required in this area, as part of the further validation of our approach, in particular, the degree of deviation with renal impairment, which is known to be associated with lithium treatment (Wiuff et al., 2024).

It is of note that the performance of the one-compartment models (ours and Pepin) was the most affected by the removal of the four outliers, which we consider to be an artifact of the construction of the other models. Keck uses a flat estimate (weight) whilst Zetin, Chiu, Terao and Abou-Auda models are all derived from multilinear regression from a population of patients – deviations from expected (such as a serum sample taken outside the desired time interval post dose, with respect to the original population) are skewed towards the mean of the original sample, suppressing the effect of outliers. We suggest that this analysis provides additional evidence to highlight the sensitivity of our model – an important consideration for lithium dosing.

Our model also demonstrated a high PPV of 0.87, suggesting that the model performs well in identifying patients who are within the therapeutic range and may be considered a reliable tool for confirming adequate lithium levels when a positive prediction is made. In contrast, the NPV is considerably lower, at 0.125, implying that most negative predictions (i.e. predicted to be out of range) are false negatives, where the actual serum levels are within the therapeutic range. This low NPV should be interpreted with caution due to the potential influence of patient adherence, limiting the model’s reliability in identifying subtherapeutic cases. Inconsistent compliance with prescribed dosing regimens may lead to apparent discrepancies between predicted and observed levels. For example, in clinical practice, transient adherence shortly prior to blood sampling can result in therapeutic serum levels even in individuals with generally poor compliance.

### Limitations and future work

While the mean error in our one-compartment model was very low (10 mg), the standard deviation was close to one whole tablet (148 mg). A similar level of error was seen in the non-linear mixed effects popPK models; thus, we presume that the error observed was systematic in nature. We suspect that the sampling time relative to the dose is a major factor and will seek to explore this in future studies, which will exert tighter control on the exact administration and sampling time. While we assumed that samples were withdrawn exactly 12 hours post dose, although sampling times were fixed, subjects may not have taken the tablets at the exact time specified. Further, serum lithium sampling at 12 hours post dose is standard in practice, but for a once-daily regimen, it does not represent a trough level. Ideally, a single pharmacokinetic serum sample should be taken just prior to the next dose (Stroud et al., 2001), to minimise variability within and between subjects. Lastly, the BLISS cohort had lithium levels accurate only to increments of 0.1 mmol/L (standard practice for that laboratory), and taking an example that minimises this error, a lithium level rounded to 1 mmol/L from 0.95 mmol/L could produce an error of about 5% (60 mg).

A further limitation of the work is that our model has so far only been tested in two independent research samples, in which all subjects were taking lithium long-term and euthymic at the time of investigation; thus, we are unable to address the potential effects of mood state. Both studies also included only working-age adults without significant physical health co-morbidities, which can affect pharmacokinetics. Most notably, the eGFR of all subjects was above 60 mL/min. Clinically relevant elimination of lithium is mediated by the kidneys, and therefore, some previous predictive models have sought to link lithium elimination rate with eGFR. The eGFR extrapolates a single blood plasma measurement of creatinine to estimate kidney function, which must then be scaled to estimate the elimination rate of lithium. We recognise that our finding of no relationship between predictive error and the submitted covariates (weight, height, age, serum creatinine, creatinine clearance and sex) is likely due to the small sample size and because the study actively excluded patients at extremes of age and with eGFR of ⩽ 60 mL/min. We suggest that the relationship with eGFR may be more apparent in a targeted study of patients with lower eGFRs, an important factor that is likely relevant for patients receiving lithium where the eGFR is known to decline faster than normal (Tondo et al., 2017). This is also consummate with the recognition that eGFR is less valuable at the extremes of age and weight. Understanding these factors will enable us to modify the elimination term, which we set to 24 hour, the mid-point of lithium’s summary of product characteristics (Electronic Medicines Compendium, 2025). Due to the location of the research centres, ethnicity was primarily White European so future work will test this model in a larger, more diverse patient population and explore the relationship between renal function and lithium clearance, particularly when eGFR is <60 mL/min. We propose that understanding the relationships of these variables on serum lithium concentrations will increase model precision, and ultimately confidence in application in practice.

### Future developments

We have explored proof-of-principle approaches to embed the one-compartment equation in a calculator within commonly available desktop software (Microsoft Excel) and a clinician-facing user-friendly Web application. As well as delivering increased accuracy in predicting target lithium dose for a patient, our pharmacokinetic first principles approach can be customised to a variety of clinical contexts, including a range of lithium formulations by conversion from salt dose into mmol of lithium, dosage regimens (once or twice daily) and serum lithium target ranges. It should be noted that our model assumed measurement at steady state (typically up to 5 days for lithium), so it cannot predict serum levels before that point and cannot be used when the dose regimen is asymmetrical (such as twice daily dosing with 600 mg morning and 400 mg at night). Nevertheless, this approach has numerous advantages and applications that once validated, could empower clinicians in their decision-making regarding lithium titration. We have undertaken patient and public involvement activities to obtain the views of relevant stakeholders on its clinical application, with positive feedback from people with lived experience of using and prescribing lithium.

Another potential application for this calculator is the estimation (or back-projection) of 12-hour levels based on lithium levels from different sampling times, by simple algebraic rearrangement of our formula also allows the estimation (or back-projection), which would be valuable for clinical practice where flexibility in lithium monitoring would be welcome by patients and services. Whilst a simple equation for estimating the 12-hour serum lithium concentration using levels measured at alternative time points has recently been reported (Köhler-Forsberg, 2025), this was developed using regression methods, and future work could compare the accuracy of the two methods. We are also interested in determining the potential of such equations to predict the necessary dose reductions required to reduce the serum lithium concentration by a desired amount, which could be of value during lithium discontinuation, where sudden cessation may increase the likelihood of relapse.

## Conclusion

Accurate determination of lithium dose has been a longstanding research objective with the potential to directly influence clinical care to patient’s advantage. Our model has shown benchmark predictive capacity in two independent cohorts of patients enrolled in research studies in the United Kingdom and Germany. Whilst further validation in larger and more diverse clinical populations is required, its flexibility and scope for integration into a desktop tool or application have the potential for direct impact on practice. If proven robust, such a tool should enable clinicians to predict a target lithium dose during consultations and may aid the interpretation of serum lithium levels taken outside of the 12-hour window. This increases confidence in dose titration (so long as serum sampling respects steady-state time intervals) and facilitates collaborative planning of initial lithium treatment plans if the anticipated dose and number of incremental steps can be predicted. This in turn has the potential to reduce the individual and economic costs associated with lithium monitoring and, from a global psychiatry perspective, facilitate lithium prescribing in resource-poor settings where ideal monitoring may not be possible.

## Supplemental Material

sj-docx-1-jop-10.1177_02698811251378508 – Supplemental material for A one-compartment model provides benchmark Lithium dose prediction Supplemental material, sj-docx-1-jop-10.1177_02698811251378508 for A one-compartment model provides benchmark Lithium dose prediction by Oisín N. Kavanagh, Elliot Asprey, Katinka A. Edelmann, Philipp Ritter, David A. Cousins and Victoria C. Wing in Journal of Psychopharmacology
