# Supplementary material for: A one-compartment model provides benchmark Lithium dose prediction
Source: J Psychopharmacol. 2025 Oct 29;40(6):964–71. doi: 10.1177/02698811251378508 (PMC13351109; doi:10.1177/02698811251378508)

Supplemental information

# A one compartment model provides benchmark Lithium dose prediction

Oisín N. Kavanagh*^, †^, Elliot Asprey^†, ‡^, Katinka A. Edelmann*^◊^*, Philipp Ritter*^◊,^* ^¶^, David A. Cousins^‡, §, #^, Victoria C. Wing^‡, #^

Figure S1 highlights that percent predictive error (predicted vs. actual lithium concentration) is randomly distributed when plotted against age, weight, sex, height, dose, serum creatinine and creatinine clearance, this may be related to the small sample size.

**Figure S1**. Predictive error as a function of BLISS cohort parameters.


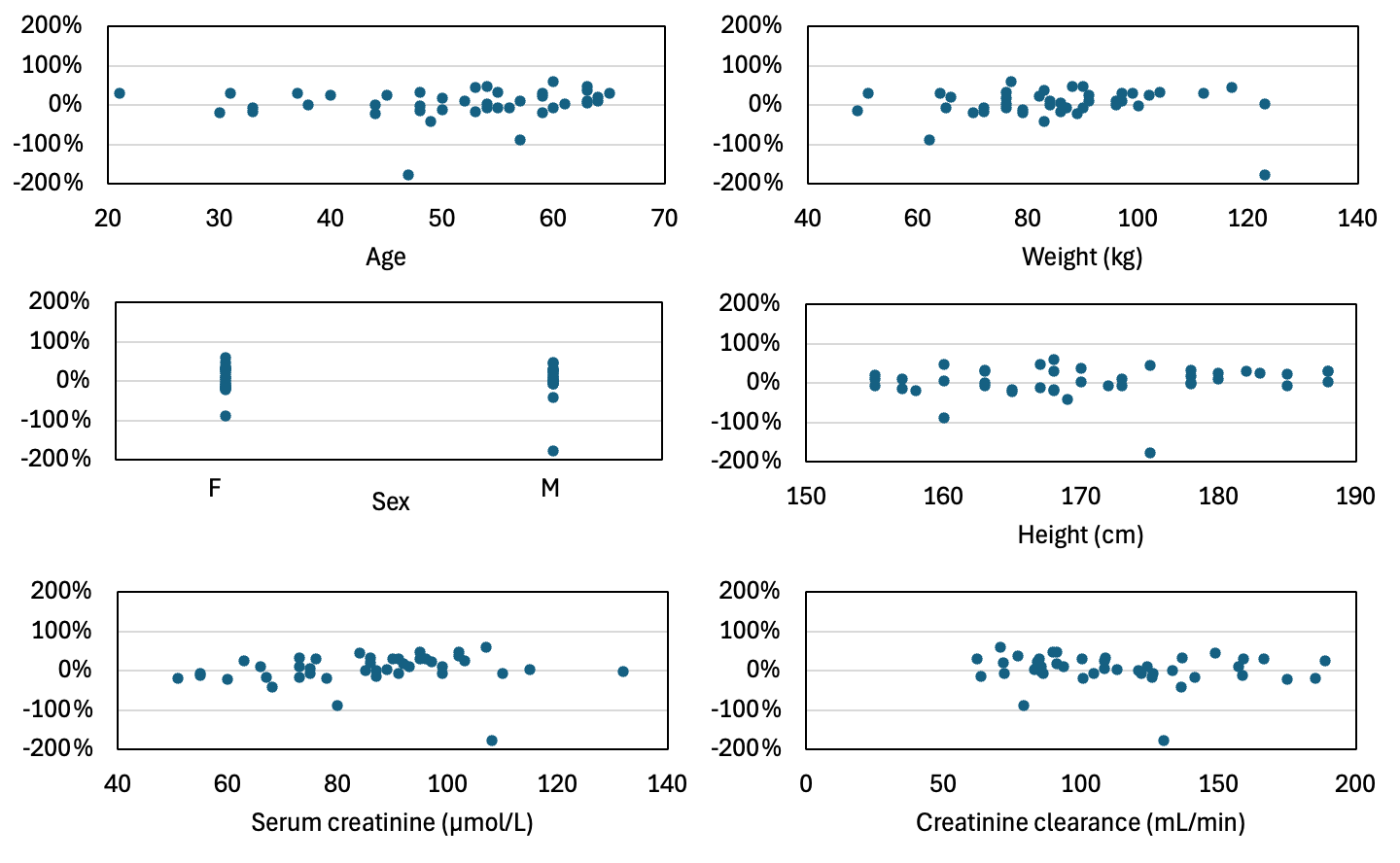


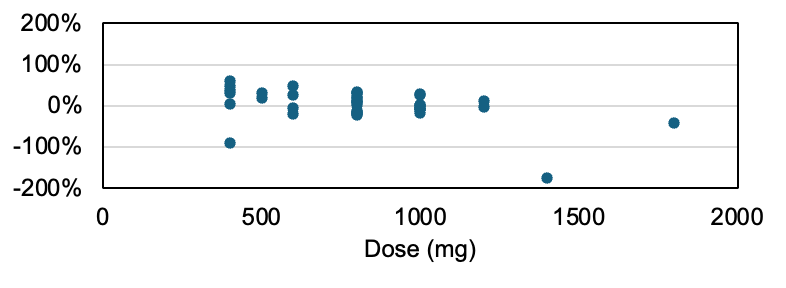

Supplement: sj-docx-1-jop-10.1177_02698811251378508 – Supplemental material for A one-compartment model provides benchmark Lithium dose prediction [file sj-docx-1-jop-10.1177_02698811251378508.docx]
